# The efficacy and safety of beta-blockers versus cyanoacrylate injection for gastric variceal bleeding: A protocol for systematic review and meta-analysis

**DOI:** 10.1097/MD.0000000000026039

**Published:** 2021-05-28

**Authors:** Yubao Sun, Sheng Li, Feng Li

**Affiliations:** a Department of Emergency Surgery; b Department of Radiology, Weifang People's Hospital, Shandong 261041, China.

**Keywords:** beta-blockers, cyanoacrylate, gastric variceal bleeding, meta-analysis, protocol

## Abstract

**Background:**

The benefit of beta-blockers for secondary prophylaxis of gastric variceal bleeding has limited evidence. Therefore, a systematic review and meta-analysis was conducted to systematically analyze and compare the effect of beta-blockers versus cyanoacrylate injection for patients with gastric variceal bleeding.

**Methods:**

The Preferred Reporting Items for Systematic Reviews and Meta-Analyses reporting guidelines will be followed to conduct the present meta-analysis. From the inception to June 2021, the Web of Science, EMBASE, PubMed, and Cochrane Library electronic databases will be searched using the key phrases “beta-blockers,” “cyanoacrylate,” and “gastric variceal bleeding” for all relevant English-language trials. Study included in our meta-analysis has to meet the following criteria: observational or randomized controlled trial focusing on assessing the effectiveness of beta-blockers and cyanoacrylate injection for gastric variceal bleeding; the following outcome measures are reported: bleeding from gastric variceal, overall mortality, bleed related mortality, and complications.

**Results:**

This study expects to provide credible and scientific evidence for the efficacy and safety of beta-blockers versus cyanoacrylate injection for patients with gastric variceal bleeding.

**Registration number:**

10.17605/OSF.IO/CPV9T.

## 1 Introduction

Gastric variceal bleeding is more severe, life-threatening, requiring more blood transfusions, and associated with higher mortality and morbidity compared with esophageal variceal hemorrhage. The treatment of bleeding from ruptured gastric variceal is challenging and requires expertise, as large amounts of rebleeding may occur.^[1]^ Treatment of varicose veins by cyanoacrylate injection via standard gastroscopy has a higher rate of hemostasis and a lower rate of rebleeding compared with band ligation or sclerotherapy, but may be associated with adverse events such as pulmonary embolism, bleeding, fever, chest pain, and even death. In addition, endoscopic injection of cyanoacrylate has been shown to damage the endoscopic working channel. Furthermore, complete varicose occlusion may be difficult to identify during surgery, and additional treatment may be required.^[2,3]^

Beta-blockers have been reported to reduce the risk of esophageal variceal rebleeding by 40% and the risk of death by 20%. However, it is not known whether it can prevent bleeding from ruptured gastric varices.^[4,5]^ In previous studies, repeated gastric variceal occlusion appeared to be more effective than beta-blockers in preventing and improving survival in patients with bleeding from gastric variceal rupture.^[6,7]^ However, the benefit of beta-blockers for secondary prophylaxis of gastric variceal bleeding has limited evidence. Therefore, a systematic review and meta-analysis was conducted to systematically analyze and compare the effect of beta-blockers versus cyanoacrylate injection for patients with gastric variceal bleeding.

## 2 Materials and methods

### 2.1 Search strategy

The Preferred Reporting Items for Systematic Reviews and Meta-Analyses reporting guidelines will be followed to conduct the present meta-analysis. From the inception to June 2021, the Web of Science, EMBASE, PubMed, and Cochrane Library electronic databases will be searched using the key phrases “beta-blockers,” “cyanoacrylate,” and “gastric variceal bleeding” for all relevant English-language trials. Moreover, references cited by the relevant sources are also hand-searched to identify any additional articles that could not be found in our database query. Ethical approval and patient consent are not required because this study was conducted based on previous studies. The systematic review protocol has been registered on Open Science Framework registries with registration number 10.17605/OSF.IO/CPV9T.

### 2.2 Eligibility criteria

Study included in our meta-analysis has to meet the following criteria: observational or randomized controlled trial (RCT) focusing on assessing the effectiveness of beta-blockers and cyanoacrylate injection for gastric variceal bleeding; the following outcome measures are reported: bleeding from gastric variceal, overall mortality, bleed related mortality, and complications. Duplicate reports and conference abstracts are excluded. Case reports, biochemical trials, letters, and reviews are also eliminated. Two independent authors screen the titles and abstracts of potentially relevant studies to determine their eligibility based on the criteria.

### 2.3 Data extraction

The method of data extraction will follow the approach outlined by the *Cochrane Handbook for Systematic Reviews of Interventions*. Two independent authors extract the following descriptive raw information from the selected studies: study characteristics such as author, publication year, study design; patient demographic details such as patients’ number, average age, body mass index, and gender ratio. The primary outcome is bleeding from the gastric variceal. Secondary outcome measures include overall mortality, bleed-related mortality, and complications. Where disagreement in the collection of data occurs, this is resolved through discussion. If the data are missing or cannot be extracted directly, we will contact the corresponding authors to ensure that the information is integrated. Otherwise, we calculate them with the guideline of *Cochrane Handbook for Systematic Reviews of Interventions*. If necessary, we will abandon the extraction of incomplete data.

### 2.4 Statistical analysis

Review Manager software (v 5.3; Cochrane Collaboration) is used for the meta-analysis. Extracted data are entered into Review Manager by the first independent author and checked by the second independent author. Risk ratio with a 95% confidence interval or standardized mean difference with 95% CI is assessed for dichotomous outcomes or continuous outcomes, respectively. The heterogeneity is assessed using the *Q* test and *I*^2^ statistic. An *I*^2^ value of <25% is chosen to represent low heterogeneity and an *I*^2^ value of >75% to indicate high heterogeneity. All outcomes are pooled on a random-effect model. A *P* value of <0.05 is considered to be statistically significant.

### 2.5 Quality assessment

The Cochrane risk of bias tool is independently used to evaluate the risk of bias of included RCTs by 2 reviewers. The quality of RCTs is assessed using the following 7 items: random sequence generation, allocation concealment, blinding of participants and personnel, blinding of outcome assessment, incomplete outcome data, selective reporting, and other bias. A modified version of the Downs and Black tool is adopted to evaluate the quality of nonrandomized cohort studies. The modified version consists of 27 items with a total possible score of 29. A score of ≥75% indicates high quality, 60%–74% indicates moderate quality, and ≤60% low quality. Two investigators independently evaluate included studies on the 27 criteria, with any discrepancies resolved by a third independent reviewer. Kappa values are used to measure the degree of agreement between the 2 reviewers and are rated as follows: fair, 0.40 to 0.59; good, 0.60 to 0.74; and excellent, 0.75 or more.

## 3 Discussion

The benefit of beta-blockers for secondary prophylaxis of gastric variceal bleeding has limited evidence. Therefore, a systematic review and meta-analysis was conducted to systematically analyze and compare the effect of beta-blockers versus cyanoacrylate injection for patients with gastric variceal bleeding. The results of this research will be delivered in a peer-reviewed journal. This study expects to provide credible and scientific evidence for the efficacy and safety of beta-blockers versus cyanoacrylate injection for patients with gastric variceal bleeding.

## Author contributions

**Conceptualization:** Sheng Li, Feng Li.

**Data curation:** Yubao Sun, Sheng Li.

**Formal analysis:** Yubao Sun, Sheng Li.

**Funding acquisition:** Feng Li.

**Investigation:** Yubao Sun, Sheng Li.

**Methodology:** Yubao Sun, Sheng Li.

**Project administration:** Feng Li.

**Resources:** Feng Li.

**Software:** Yubao Sun, Sheng Li.

**Supervision:** Feng Li.

**Validation:** Yubao Sun, Sheng Li.

**Visualization:** Yubao Sun, Sheng Li.

**Writing – original draft:** Yubao Sun.

**Writing – review & editing:** Feng Li.

## References

[1] Chen WC Hsin IF Chen PH Hsu PI. Addition of carvedilol to gastric variceal obturation does not decrease recurrence of gastric variceal bleeding in patients with cirrhosis. Clin Gastroenterol Hepatol 2019;17:2356–563. e2. 30772583 10.1016/j.cgh.2019.02.021

[2] Choi MH Kim YS Kim SG. The secondary prophylactic efficacy of beta-blocker after endoscopic gastric variceal obturation for first acute episode of gastric variceal bleeding. Clin Mol Hepatol 2013;19:280–7. 24133666 10.3350/cmh.2013.19.3.280 PMC3796678

[3] Mishra SR Sharma BC Kumar A Sarin SK. Primary prophylaxis of gastric variceal bleeding comparing cyanoacrylate injection and beta-blockers: a randomized controlled trial. J Hepatol 2011;54:1161–7. 21145834 10.1016/j.jhep.2010.09.031

[4] Mishra SR Sharma BC Kumar A Sarin SK. Endoscopic cyanoacrylate injection versus beta-blocker for secondary prophylaxis of gastric variceal bleed: a randomised controlled trial. Gut 2010;59:729–35. 20551457 10.1136/gut.2009.192039

[5] Kumar A Mishra SR Sharma P Sharma BC Sarin SK. Clinical, laboratory, and hemodynamic parameters in portal hypertensive gastropathy: a study of 254 cirrhotics. J Clin Gastroenterol 2010;44:294–300. 19730114 10.1097/MCG.0b013e3181b37ea1

[6] Evrard S Dumonceau JM Delhaye M Golstein P Devière J Le Moine O. Endoscopic histoacryl obliteration vs. propranolol in the prevention of esophagogastric variceal rebleeding: a randomized trial. Endoscopy 2003;35:729–35. 12929019 10.1055/s-2003-41581

[7] Wu CY Yeh HZ Chen GH. Pharmacologic efficacy in gastric variceal rebleeding and survival: including multivariate analysis. J Clin Gastroenterol 2002;35:127–32. 12172356 10.1097/00004836-200208000-00002

